# Application of Generalized Composite Multiscale Lempel–Ziv Complexity in Identifying Wind Turbine Gearbox Faults

**DOI:** 10.3390/e23111372

**Published:** 2021-10-20

**Authors:** Xiaoan Yan, Daoming She, Yadong Xu, Minping Jia

**Affiliations:** 1 School of Mechatronics Engineering, Nanjing Forestry University, Nanjing 210037, China; 2 School of Mechanical Engineering, Jiangsu University, Zhenjiang 212013, China; 1000005461@ujs.edu.cn; 3 School of Mechanical Engineering, Southeast University, Nanjing 211189, China; ydxu@seu.edu.cn (Y.X.); mpjia@seu.edu.cn (M.J.)

**Keywords:** morphological filtering, multiscale Lempel–Ziv complexity, softmax, wind turbine gearbox, fault diagnosis

## Abstract

Wind turbine gearboxes operate in harsh environments; therefore, the resulting gear vibration signal has characteristics of strong nonlinearity, is non-stationary, and has a low signal-to-noise ratio, which indicates that it is difficult to identify wind turbine gearbox faults effectively by the traditional methods. To solve this problem, this paper proposes a new fault diagnosis method for wind turbine gearboxes based on generalized composite multiscale Lempel–Ziv complexity (GCMLZC). Within the proposed method, an effective technique named multiscale morphological-hat convolution operator (MHCO) is firstly presented to remove the noise interference information of the original gear vibration signal. Then, the GCMLZC of the filtered signal was calculated to extract gear fault features. Finally, the extracted fault features were input into softmax classifier for automatically identifying different health conditions of wind turbine gearboxes. The effectiveness of the proposed method was validated by the experimental and engineering data analysis. The results of the analysis indicate that the proposed method can identify accurately different gear health conditions. Moreover, the identification accuracy of the proposed method is higher than that of traditional multiscale Lempel–Ziv complexity (MLZC) and several representative multiscale entropies (e.g., multiscale dispersion entropy (MDE), multiscale permutation entropy (MPE) and multiscale sample entropy (MSE)).

## 1. Introduction

Wind turbines are widely used in the power system field, and are mainly composed of an impeller, rotor, gearbox, generator, bearing and coupling. The gearbox is one of the parts of the wind turbine most vulnerable to damage. The safe and steady operation of wind turbine gearboxes is directly related to the health condition of the whole mechanical system [1]. When a wind turbine gearbox exhibits local faults, the generated vibration signal is nonlinear and non-stationary. Furthermore, the generated vibration signal contains complicated and coupled vibration characteristic information (e.g., the periodic impulse, multiple harmonic interference and environment noise of signal transmission path), which indicates that it is difficult to identify wind turbine gearbox faults by common methods [2]. Therefore, to ensure the safe and steady operation of wind turbines, it is of great significance to develop a new and effective method to identify the local faults of wind turbine gearboxes.

Many methods have been successfully applied for diagnosing faults of contemporary wind turbine gearbox, such as wavelet transform (WT) [3], ensemble empirical mode decomposition (EEMD) [4], empirical wavelet transform (EWT) [5], variational mode decomposition (VMD) [6], minimum entropy deconvolution (MED) [7] and their improved versions [8]. However, these methods require considerable prior knowledge and experience of fault diagnosis, especially for the skilled calculation of fault characteristic frequencies; thus, they are not suitable for ordinary workers without practical experience. To solve this problem, an effective and popular technique is the intelligent fault diagnosis method, where the fault feature extraction is its most critical step [9]. At present, multiscale complexity index-based methods have been proven effective for fault feature extraction and have attracted more and more attention in the intelligent fault diagnosis of rotating machinery. Common complexity indexes have Lempel–Ziv complexity (LZC) [10], sample entropy (SE) [11], permutation entropy (PE) [12], dispersion entropy (DE) [13] and their multiscale versions [14]. LZC has fewer parameters compared with other complexity indexes (e.g., SE, PE and DE); therefore, it is widely used in many fields (e.g., biological signal and mechanical vibration signal analysis). Hu et al. [15] used LZC and its variants to characterize the irregularity and uncertainty of biological signals. Bai et al. [16] adopted the ordinal patterns and LZC for quantifying and describing the dynamical changes of electroencephalogram (EEG) data. Borowska [17] introduced a method named multiscale permutation Lempel–Ziv complexity (MPLZC) to evaluate the complexity of EEG signals in different temporal scales. Cui et al. [18] combined the double-dictionary matching pursuit and LZC index to realize fault extent evaluation of rolling element bearing. Yin et al. [19] used symbolic aggregate approximation and LZC to extract fault information and finish bearing fault diagnosis. Xia et al. [20] utilized the improved Hilbert vibration decomposition (HVD) and LZC to assess early damage severity for rolling bearings. Yu et al. [21] combined multiscale Lempel–Ziv complexity (MLZC) and the Mahalanobis distance criterion to identify the fault types of rolling bearings. Hong et al. [22] used LZC and continuous wavelet transform to assess the fault severity of rolling element bearings. Unfortunately, the abovementioned LZC methods have some disadvantages. On the one hand, some of the above LZC methods describe signal complexity only at a single scale, which indicates that the feature information of other scales is ignored, i.e., some of the above LZC methods cannot comprehensively extract the useful feature information. On the other hand, in the coarse-grained process of the abovementioned MLZC method, the data length of the generated coarse-grained time series will be shortened as the scale factor increases; thus, the accuracy of feature extraction will be affected. Therefore, to avoid the calculation deviation of MLZC brought by data length shortening and improve the integrality and veracity of fault feature extraction, by integrating generalized composite coarse-grained process into LZC, this paper proposes a new complexity index named generalized composite multiscale Lempel–Ziv complexity (GCMLZC) to extract more accurately and efficiently fault feature information and identify fault categories.

Of particular note, because wind turbine gearboxes operate in harsh and variable conditions, the periodic impulse features of vibration signals induced by wind turbine gearbox faults will be obscured by noise interference, which means that it is necessary to preprocess the raw vibration signal collected from wind turbine gearboxes before feature extraction. Morphological filtering (MF) is a nonlinear signal processing method containing the structuring element (SE), which has been successfully applied in signal noise reduction and is receiving increasing attention in mechanical fault diagnosis [23]. However, the noise reduction ability of existing single-scale MF methods (e.g., the dilation, erosion, opening and closing operator) is finite, and the scale selection of the structuring element (SE) of MF highly depends on human experience [24]. Hence, to address this issue, by combining the merits of multiscale morphological analysis and convolution operation in noise reduction, this paper presents a morphological convolution filtering technique named multiscale morphological-hat convolution operator (MHCO) to preprocess the collected original vibration signal, where the SE scale is determined automatically by introducing the assisted index named the signal characteristic frequency-to-noise ratio (SCFNR). In brief, main contributions of this paper include:

(1) Morphological convolution filtering with a multiscale morphological-hat convolution operator (MHCO) is presented through integrating convolution operation into morphological filtering, which can improve signal noise reduction ability;

(2) A novel complexity index called generalized composite multiscale Lempel–Ziv complexity (GCMLZC) is proposed by combining the generalized composite coarse-grained process and LZC, which can obtain more accurate and useful fault features;

(3) An intelligent fault diagnosis scheme for wind turbine gearboxes based on MHCO and GCMLZC is proposed;

(4) The effectiveness and superiority of the proposed method are validated by the experimental and engineering data analysis.

This paper is organized as follows. Section 2 describes the concept of morphological convolution filtering. Section 3 introduces the Lempel–Ziv complexity, multiscale Lempel–Ziv complexity and generalized composite multiscale Lempel–Ziv complexity. In addition, in Section 3, the superiority of generalized composite multiscale Lempel–Ziv complexity is validated by using simulation signals. Section 4 illustrates the specific procedure of the proposed fault diagnosis scheme. The effectiveness of the proposed method is proved in Section 5. The conclusions are given in Section 6.

## 2. Morphological Convolution Filtering

### 2.1. Morphological Filtering

Morphological filtering (MF) is a nonlinear signal processing method, which can effectively match and capture the details of non-stationary signals by using a probe named the structuring element (SE). Morphological filtering usually consists of four basic morphological operators (i.e., the dilation, erosion, opening and closing). If x(n)(n=0,1,2,⋯,N−1) is a one-dimensional discrete signal, g(m)(m=0,1,2,⋯,M−1) is the structuring element and *N* >> *M*, the morphological dilation, erosion, opening and closing operators are defined as:

(1) (x⊕g)(n)=max{x(n−m)+g(m)}

(2) (xΘg)(n)=min{x(n+m)−g(m)}

(3) (x∘g)(n)=(xΘg⊕g)(n)

(4) (x•g)(n)=(x⊕gΘg)(n)

where ⊕, Θ, ∘ and • represent the dilation, erosion, opening and closing operation, respectively. On the one hand, through the cascading of the four abovementioned basic morphological operators, the opening–closing (OC) and closing–opening (CO) operator are expressed as:

(5) OC(x(n))=(x∘g•g)(n)

(6) CO(x(n))=(x•g∘g)(n)

On the other hand, through the arithmetic operation of four basic morphological operators, two kinds of combined morphological operators (i.e., morphological gradient operator and morphological average-hat operator) are expressed as:

(7) GDE(x(n))=(x⊕g)(n)−(xΘg)(n)

(8) GCO(x(n))=(x•g)(n)−(x∘g)(n)

(9) GCOOC(x(n))=CO(x(n))−OC(x(n))

(10) $$AHDE(x(n))=x(n)-\frac{\left(x⊕g)(n)+(x\Theta g)(n\right)}{2}$$

(11) $$AHCO(x(n))=x(n)-\frac{\left(x•g)(n)+(x\circ g)(n\right)}{2}$$

(12) $$AHCOOC(x(n))=x(n)-\frac{CO(n)+OC(n)}{2}$$

where three operators (i.e., GDE, GCO and GCOOC) belong to the morphological gradient operator, whereas another three operators (i.e., AHDE, AHCO and AHCOOC) belong to the morphological average-hat operator.

### 2.2. Morphological Convolution Filtering

Considering the advantages of convolution operation in signal noise reduction, morphological convolution filtering, termed as a morphological gradient convolution operator (MGCO), was proposed by Li et al. [25] in 2018, which is defined as follows:

(13) MGCO(x(n))=GCO(x(n))∗GCOOC(x(n))

where the asterisk, *, denotes the convolution operation.

Inspired by the concept of the MGCO, an alternative morphological convolution filtering method hailed as the morphological-hat convolution operator (MHCO) is formulated as follows:

(14) MHCO(x(n))=AHCO(x(n))∗AHCOOC(x(n))

Due to fault feature information of practical wind turbine gearboxes, vibration signals are distributed over a wide frequency band; thus, excavating gear fault features by only using single-scale morphological filtering is inadequate. Therefore, the multiscale morphological-hat convolution operator is further defined as follows:

(15) $$MHCO(x{\left(n\right)}_{\lambda g})=AHCO(x{\left(n\right)}_{\lambda g})*AHCOOC(x{\left(n\right)}_{\lambda g})$$

where g is the single-scale SE, λg is the multiscale SE at scale λ, which can be obtained by dilation operation of λ−1 times of g. Specifically, the multiscale SE λg can be expressed as follows [26]:

(16) $$\lambda g=\underset{\lambda -1\begin{array}{c} times \end{array}}{\underset{︸}{g⊕g⊕\cdots ⊕g}}=\underset{\lambda -1\begin{array}{c} times \end{array}}{\underset{︸}{((g⊕\cdots ⊕g)⊕g)⊕g}}$$

Through the introduction and application of multiscale SE λg, the proposed morphological convolution filtering (MHCO) process can successfully realize multi-resolution signal analysis and more accurately extract the fault feature information of wind turbine gearbox vibration signals than traditional morphological filtering. Theoretically, if fault feature frequency can be extracted by AHCO and AHCOOC, MHCO can also extract the same fault feature frequency, and the amplitude extracted by MHCO is larger than that AHCO and AHCOOC. Nevertheless, the existing related studies have shown that selection of the SE scale has a certain impact on the noise reduction performance of morphological filtering, which indicates that an effective selection strategy needs to be introduced in morphological convolution filtering.

At present, the research on selection method of SE scale of morphological filtering mainly focuses on two aspects. Firstly, many intelligent optimization algorithms (e.g., particle swarm optimization, genetic algorithm and differential evolution algorithm) are adopted to automatically select the SE scale of morphological filtering, but they will consume a lot of computational time under the iteration process. Secondly, some sensitive indexes (e.g., kurtosis, signal-to-noise ratio (SNR) [27] and fault feature ratio (FFR) [28]) are presented for the auxiliary selection of the SE scale of morphological filtering. Although these indexes had been proven effective in selecting the SE scale of morphological filtering, they also suffer from some disadvantages. For instance, kurtosis can detect periodic impulse characteristics of the vibration signal, but it is susceptible to random impulses with a large amplitude. Moreover, due to the influence of a complex environment, the fault signatures in the real gear vibration signal are indistinct and dispersive, which are difficult to detect through the kurtosis index. SNR can efficaciously evaluate stochastic noise interference of the vibration signal, but its robustness is weak for the impact property of the vibration signal. FFR can effectively characterize repetitive transients of the signal, but it is insensitive to stochastic noise of the vibration signal. Therefore, considering the merits and demerits of SNR and FFR, this paper introduces a synthetic sensitive indicator called the signal characteristic frequency-to-noise ratio (SCFNR) to automatically determine the optimal SE scale of MHCO, which is defined as follows [29]:

(17) $$SCFNR=10\log_{10}\frac{\sum_{i=1}^{M}S(f_{ci})}{\sum_{j=1}^{N}S(f_{j})-\sum_{i=1}^{M}S(f_{ci})}$$

where $f_{ci}$ is the *i*th fault characteristic frequency of Hilbert envelope spectrum of the original signal *x*(*n*), $S(f_{ci}),i=1,2,\cdots ,M$ is the amplitude of Hilbert envelope spectrum of the original signal *x*(*n*) at the *i*th fault characteristic frequency, $S(f_{j}),j=1,2,\cdots ,N$ is the amplitude of Hilbert envelope spectrum of the original signal *x*(*n*) at the *j*th frequency *f*, and *N* and *M* are the number of all frequencies and fault characteristic frequencies of the Hilbert envelope spectrum of the original signal *x*(*n*), respectively. The SCFNR indicator is derived from the theoretical ideas of SNR and FFR; therefore, it inherits the advantages of SNR and FFR. The greater the SCFNR indicator, the better the noise reduction performance of MHCO, i.e., MHCO has better fault feature extraction performance. Therefore, the optimal SE scale of MHCO can be determined based on the largest SCFNR value.

### 2.3. Simulation Analysis

**Figure 1:**
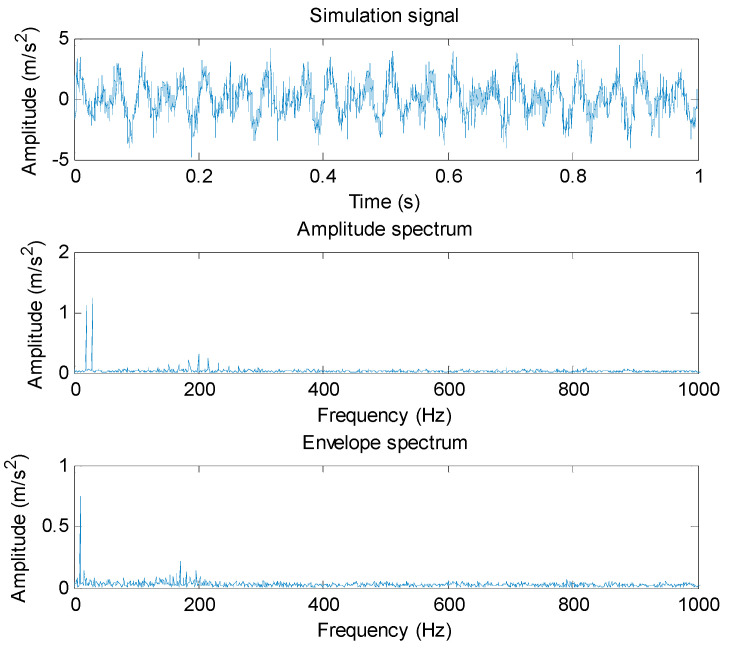
Time domain waveform, amplitude spectrum and envelope spectrum of simulation signal.

To verify the validity of MHCO in extracting periodic impulse features of vibration signals, a gear fault simulation signal *y*(*t*) is formulated as:

(18) $$\left\{\begin{array}{l} y(t)=s_{1}(t)+s_{2}(t)+r(t)\\ s_{1}(t)=2\exp(-at_{0})\cos(2\pi f_{1}t)\\ s_{2}(t)=1.1\sin(2\pi f_{2}t)+1.2\sin(2\pi f_{3}t) \end{array}\right.$$

where $t_{0}=\mathrm{mod}(k/f_{s},1/f_{o})$, k=0,1,2,⋯,2047. The simulation signal *y*(*t*) consists of three parts ($s_{1}(t)$, $s_{2}(t)$ and r(t)). $s_{1}(t)$ is a periodic impulse sequence with the amplitude of 2, carrier frequency of *f*_1_ = 200 Hz, modulation frequency (i.e., gear fault characteristic frequency) of $f_{o}$ = 16 Hz and attenuation coefficient of *a* = −100, which is used to simulate the impact signal generated by gear faults. Thus, the signal period of $s_{1}(t)$ is equal to 0.0625 s. $s_{2}(t)$ is a sinusoidal superimposed signal with the frequency of *f*_2_ = 20 Hz and *f*_3_ = 30 Hz, which is used to simulate the harmonic interference signals in gear vibration signals. r(t) is the Gaussian white noise with a signal-to-noise ratio (SNR) of 3 dB, which is used to simulate the background noise in gear vibration signal. The sampling frequency *f*_s_ and sampling length of the simulation signal *y*(*t*) are set as 2048 Hz and 2048 points, respectively. Figure 1 shows the time domain waveform, amplitude spectrum and envelope spectrum of simulation signal *y*(*t*). Seen from the spectrum of Figure 1, due to the harmonic interference and background noise, the fault feature frequency $f_{o}$ = 16 Hz cannot be extracted by using directly the amplitude spectrum and envelope spectrum.

**Figure 2:**
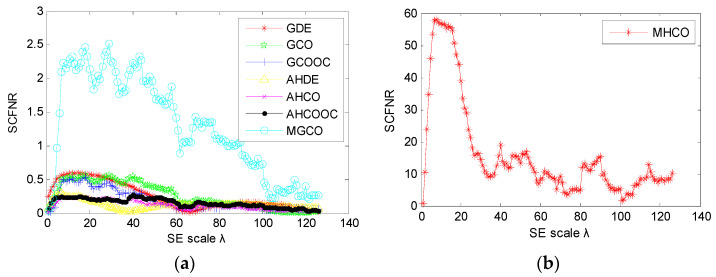
The SCFNR value of each morphological filtering at the different SE scales, (**a**) seven representative morphological filtering (i.e., GDE, GCO, GCOOC, AHDE, AHCO, AHCOOC and MGCO); (**b**) MHCO.

**Figure 3:**
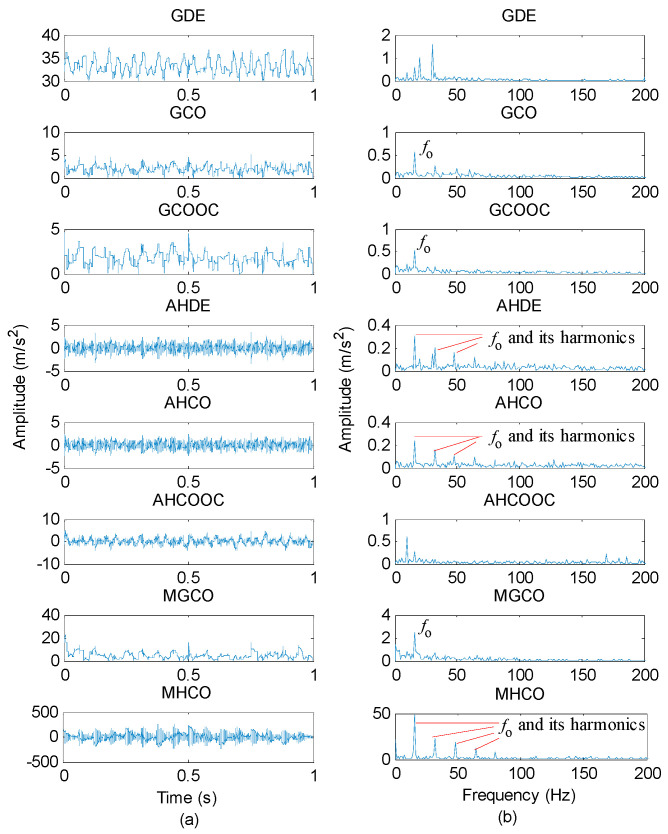
(**a**) The filtered signal obtained by different morphological filtering methods and (**b**) their corresponding envelope spectrum.

The proposed MHCO method and seven representative morphological filtering (i.e., GDE, GCO, GCOOC, AHDE, AHCO, AHCOOC and MGCO) are adopted to process the simulation signal *y*(*t*), respectively. In the comparison, all morphological filtering selected the widely used flat SE and the optimal SE scale was determined by the largest SCFNR. According to the literature [30], the maximal length of the flat SE is recommended as $\left\lfloor f_{s}/f_{o}\right\rfloor$, which represents the number of sampling points in one fault repetition period, i.e., the value of $\left\lfloor f_{s}/f_{o}\right\rfloor$ can completely cover one fault repetition period. In addition, the length L and scale λ of the flat SE satisfy the relationship L=λ+2. Therefore, the search range of the flat SE scale λ is set as 1 to $\left\lfloor f_{s}/f_{o}\right\rfloor -2$, where *f*_s_ is the sampling frequency, *f_o_* is the fault feature frequency and $\left\lfloor \cdot \right\rfloor$ denotes the down round operation. In other words, in the simulation signal, the flat SE scale λ=1,2,⋯,126. According to the SE scale selection criterion, the SCFNR of different morphological filter methods are first calculated to determine their optimal SE scale. Figure 2 plots the SCFNR value obtained by all methods at different SE scales, and Table 1 lists the largest SCFNR obtained with different methods and their corresponding optimal SE scale. Then, different methods with the optimal SE scale are used to analyze the simulation signal *y*(*t*). Figure 3 shows the filtered signal obtained by different methods and their corresponding envelope spectrum. Seen from the envelope spectrum of Figure 3, the MHCO, AHDE and AHCO can effectively extract the fault characteristic frequency $f_{o}$ = 16 Hz and its harmonics. In addition, the fault characteristic frequency $f_{o}$ can also be found in the GCO, GCOOC and MGCO, but the fault characteristic frequency $f_{o}$ is invisible in the GDE and AHCOOC. Overall, compared with the seven other methods (i.e., GDE, GCO, GCOOC, AHDE, AHCO, AHCOOC and MGCO), the amplitude of the fault characteristic frequency obtained by the proposed MHCO is the largest, which indicates that the proposed MHCO has better noise reduction performance. Therefore, it can be seen from the simulation analysis results that the proposed MHCO is effective in eliminating the noise interference of gear vibration signals.

**Figure 4:**
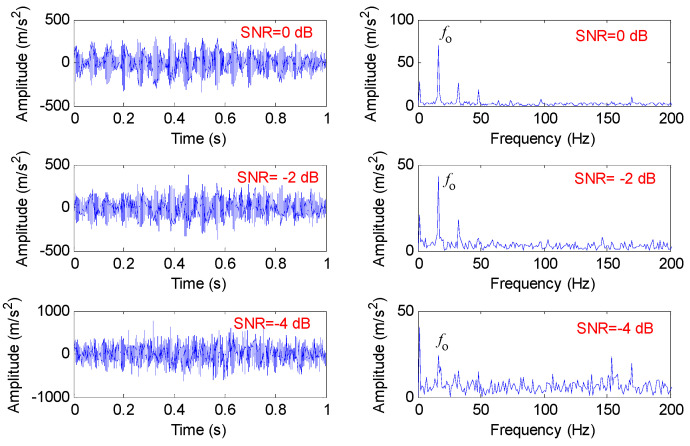
The results obtained by the MHCO method under different noise levels (i.e., SNR = 0 dB, SNR = −2 dB and SNR = −4 dB).

**Figure 5:**
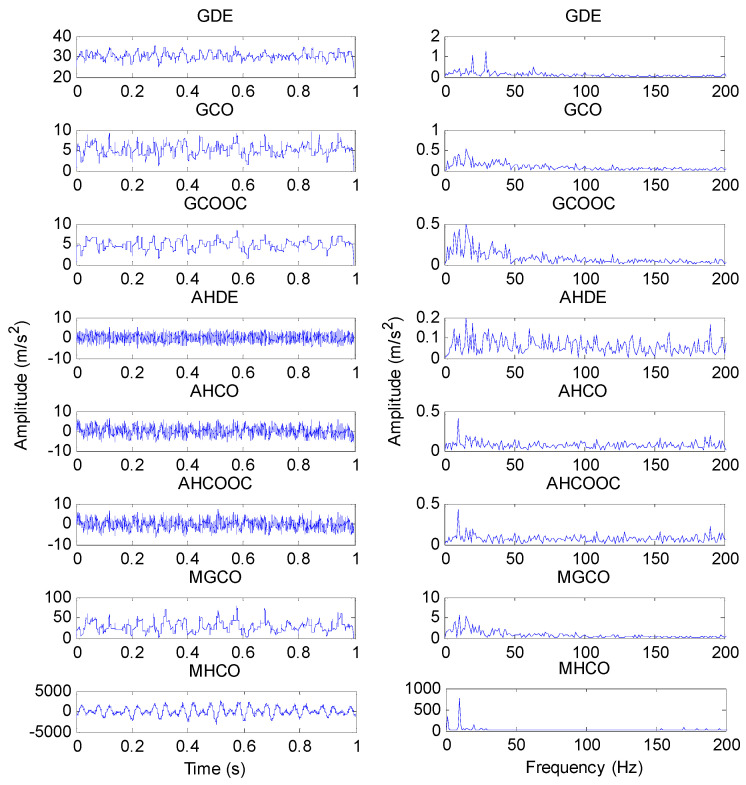
The results obtained by different morphological filtering methods under SNR = −5 dB.

To study the influence of the added noises on the MHCO method, we calculated the results of the MHCO method under different noise levels (i.e., SNR = 0 dB, SNR = −2 dB and SNR = −4 dB), as shown in Figure 4. From Figure 4, when the SNR of Gaussian white noise of gear fault simulation signal *y*(*t*) is set as 0 to −4 dB, the proposed MHCO is still effective in extracting the gear fault characteristic frequency *f_o_*. However, when the SNR of Gaussian white noise is −5 dB, the proposed MHCO and other morphological filtering cannot effectively extract the gear fault characteristic frequency *f_o_* (see Figure 5). Hence, in the first example, the limit of SNR of simulation signal is empirically considered as −5 dB.

## 3. Generalized Composite Multiscale Lempel–Ziv Complexity

To overcome the shortcoming of information loss of coarse-grained process existing in the traditional MLZC method, a new complexity index named generalized composite multiscale Lempel–Ziv complexity (GCMLZC) is presented in this section.

### 3.1. Lempel–Ziv Complexity

**Figure 6:**
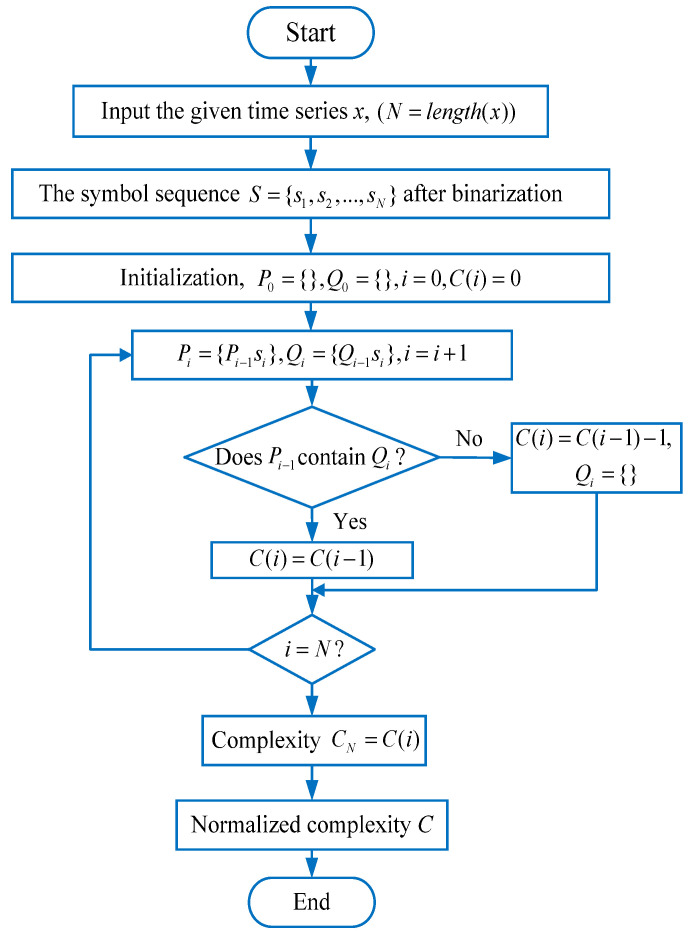
Flowchart of the LZC method.

Lempel–Ziv complexity (LZC) is a widely used tool which can effectively describe the randomness and uncertainty of time series. Figure 6 shows the flowchart of the LZC method. For a given time series $\left\{x(i),i=1,2,\cdots ,N\right\}$, the specific steps of LZC are described as follows:

(1) According to Equation (19), the binary coarse-grained analysis is used to process the original time series, i.e., according to the 0–1 binary encoding, the original time series is reconstructed to obtain the symbol sequence $S=\left\{s_{1},s_{2},\cdots ,s_{N}\right\}$. Specifically, the mean value, *T_d_*, of the original time series is first calculated; then, the points in the original time series that are greater than the mean value are assigned a value of 1, and the points that are less than the mean value are assigned a value of 0.

(19) $$s_{i}=\left\{\begin{array}{l} \begin{array}{cc} 0, & \mathrm{if}\;x(i)<T_{d} \end{array}\\ \begin{array}{cc} 1, & \mathrm{otherwise} \end{array} \end{array}\right.$$

(2) Initialize $P_{0}$ and $Q_{0}$ as the empty matrices and set the initial value i=0. At this time, the complexity C(i)=0.

(3) Perform a loop operation. Set $P_{i}=\left\{P_{i-1}s_{i}\right\}$, $Q_{i}=\left\{Q_{i-1}s_{i}\right\}$, and then judge whether $P_{i-1}$ contains $Q_{i}$. If the judgment result is “Yes”, the complexity C(i) will remain as the same value, i.e., C(i)=C(i−1). Otherwise, C(i)=C(i−1)+1,$Q_{i}=\{\}$. Notably, in this step, it will loop *N* times until the symbol sequence *S* is traversed, and the last complexity C(N) can be obtained.

(4) Normalize the complexity $C_{N}$ to obtain the final Lempel–Ziv complexity value. Specifically, for the binary symbol sequence *S*, the normalized Lempel–Ziv complexity is calculated by

(20) $$C=\frac{C_{N}\log_{2}N}{N}$$

### 3.2. Multiscale Lempel–Ziv Complexity

For a given time series $\left\{x(i),i=1,2,\cdots ,N\right\}$, the specific calculation process of MLZC can be described as follows:

(1) Using Equation (21) obtains a coarse-grained time series $y_{j}^{\left(\tau \right)}$ with the length of N/τ.

(21) $$y_{j}^{\left(\tau \right)}=\frac{1}{\tau }\sum_{i=(j-1)\tau +1}^{j\tau }x_{i},\;j=1,2,\cdots ,N/\tau$$

where τ=1,2,⋯ represents the scale factor. Apparently, when the scale factor τ=1, $y_{j}^{\left(1\right)}(j=1,2,\cdots ,N)$ amounts to the original time series.

**Figure 7:**
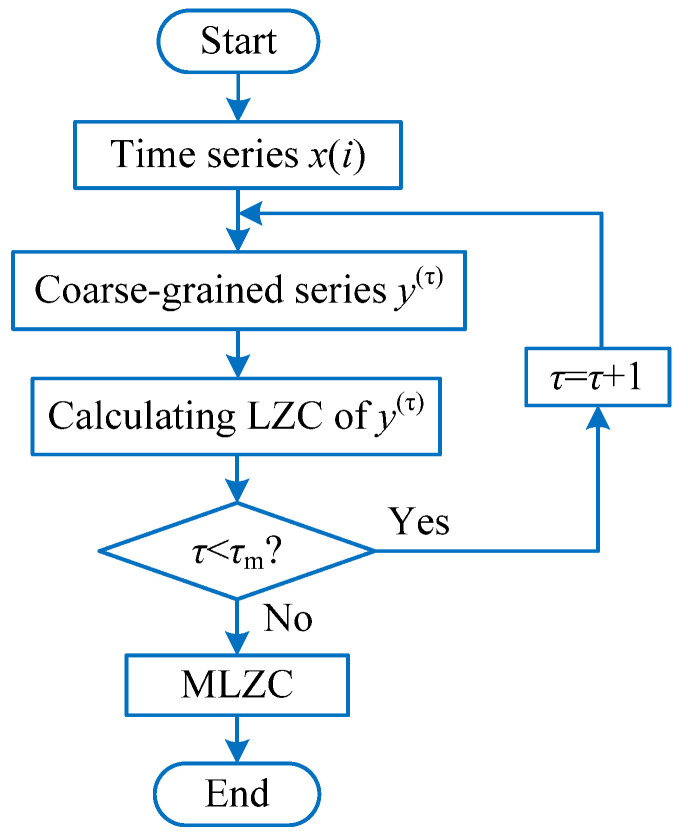
Flowchart of the MLZC method.

(2) According to Equation (22), the LZC value of each coarse-grained time series $y_{j}^{\left(\tau \right)}$ can be calculated to obtain the final multiscale Lempel–Ziv complexity.

(22) $$\mathrm{MLZC}(x,\tau )=LZC(y_{j}^{\left(\tau \right)})$$

where τ is the scale factor and LZC(•) is the operator of Lempel–Ziv complexity. Figure 7 shows the flowchart of the MLZC method.

### 3.3. Generalized Composite Multiscale Lempel–Ziv Complexity

For a given time series {x(i),i=1,2,⋯,N}, the specific calculation process of GCMLZC is given as follows:

(1) Using Equation (23) to obtain the generalized composite coarse-grained time series $y_{k}^{\left(\tau \right)}=\{y_{k,j_{1}}^{\left(\tau \right)},y_{k,j_{2}}^{\left(\tau \right)},\cdots ,y_{k,j_{\tau }}^{\left(\tau \right)}\}$. Concretely, in the GCMLZC method, when the scale factor τ = 1, one generalized composite coarse-grained time series $y_{k}^{\left(1\right)}$ can be obtained, which is equivalent to the original time series. When the scale factor τ = 2, two generalized composite coarse-grained time series $y_{k}^{\left(1\right)}$ and $y_{k}^{\left(2\right)}$ can be obtained. Nevertheless, in the MLZC method, if the scale factor τ = 2, we can only obtain one coarse-grained time series $y_{k}^{\left(2\right)}$.

(23) $$y_{k,j}^{\left(\tau \right)}=\frac{1}{\tau }\sum_{i=k+(j-1)\tau }^{k+j\tau -1}{\left(x_{i}-{\bar{x}}_{i}\right)}^{2},\;1\leq j\leq \left\lfloor \frac{N}{\tau }\right\rfloor ,\;1\leq k\leq \tau$$

where ${\bar{x}}_{i}=\frac{1}{\tau }\sum_{k=0}^{\tau -1}x_{i+k}$ and 2≤τ.

(2) For the scale factor τ, calculating the LZC value of each generalized composite coarse-grained time series $y_{k}^{\left(\tau \right)}(k=1,2,\cdots ,\tau )$.

(3) Equation (24) can be used to calculate the mean LZC value as the LZC result of the scale factor τ of the original time series.

(24) $$\mathrm{GCMLZC}(x,\tau )=\frac{1}{\tau }\sum_{k=1}^{\tau }LZC(y_{k}^{\left(\tau \right)})$$

where τ is the scale factor and LZC(•) is the operator of Lempel–Ziv complexity.

**Figure 8:**
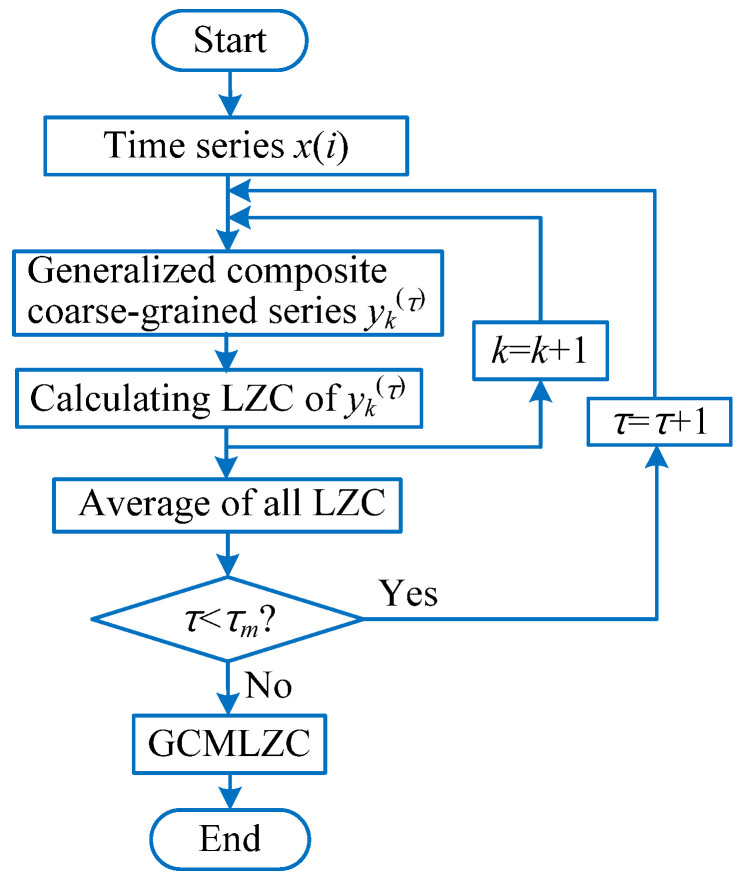
Flowchart of the GCMLZC method.

(4) Judging whether the scale factor τ reaches its maximum value $\tau_{m}$. If the scale factor $\tau <\tau_{m}$, set τ = τ + 1, return to steps (2) and (3) and continue to run the procedure until $\tau =\tau_{m}$. Otherwise, stop the circulation process and output the final results of the GCMLZC method. In other words, after performing the GCMLZC method, for the scale factor τ, a series of LZC value can be obtained. It is worth mentioning that the scale factor τ of GCMLZC method is between 2 and $\tau_{m}$. In addition, without a loss of generality, the largest scale factor $\tau_{m}$ is selected as 20, which is regarded as an empirical value. Figure 8 shows the flowchart of the GCMLZC method.

### 3.4. Comparison among LZC, MLZC and GCMLZC

**Figure 9:**
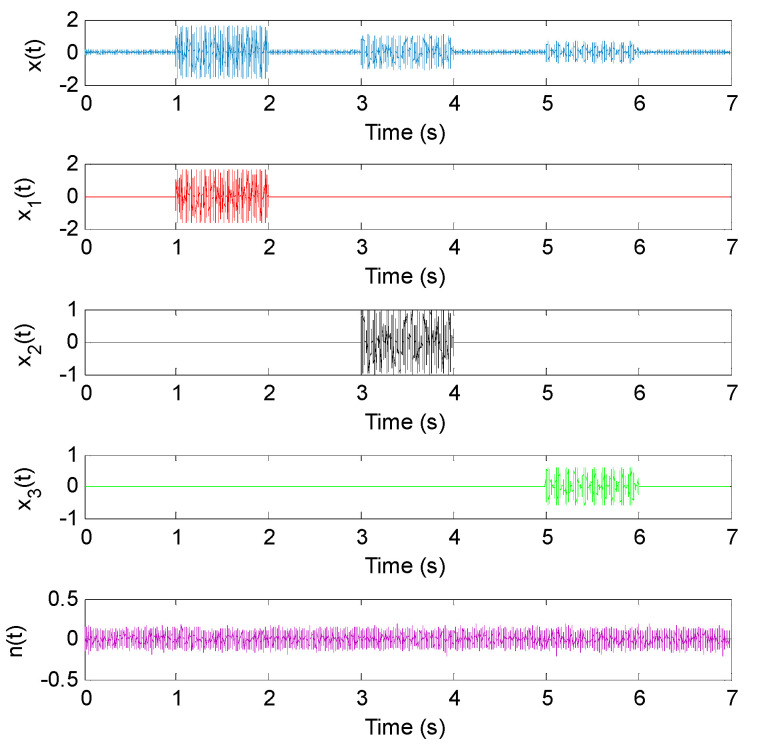
Time domain waveform of simulation signal *x*(*t*) and its corresponding components.

To show the feature extraction performance of the proposed GCMLZC method, here, one intermittent multi-component amplitude-modulated and one frequency-modulated signal *x*(*t*) are established as follows:

(25) $$\left\{\begin{array}{l} x_{1}(t)=\left\{\begin{array}{l} (0.8+0.8\sin(20\pi t))\cos(3000\pi t+2\sin(15\pi t))\begin{array}{cc} , & 1<t\leq 2 \end{array}\\ \begin{array}{cc} 0, & \mathrm{other} \end{array} \end{array}\right.\\ x_{2}(t)=\left\{\begin{array}{l} (0.5+0.5\sin(15\pi t))\cos(1500\pi t+\sin(10\pi t))\begin{array}{cc} , & 3<t\leq 4 \end{array}\\ \begin{array}{cc} 0, & \mathrm{other} \end{array} \end{array}\right.\\ x_{3}(t)=\left\{\begin{array}{l} (0.3+0.3\sin(10\pi t))\cos(600\pi t+0.5\sin(5\pi t))\begin{array}{cc} , & 5<t\leq 6 \end{array}\\ \begin{array}{cc} 0, & \mathrm{other} \end{array} \end{array}\right.\\ x(t)=x_{1}(t)+x_{2}(t)+x_{3}(t)+n(t) \end{array}\right.$$

where *n*(*t*) is the Gaussian white noise with an SNR of 32 dB. *x*_1_(*t*), *x*_2_(*t*) and *x*_3_(*t*) are used to simulate gear fault signals with different frequencies (i.e., 1500 Hz, 750 Hz and 300 Hz), respectively. The sampling frequency and sampling length of simulation signal *x*(*t*) are 5000 Hz and 35,000 points. Figure 9 shows the time domain waveform of simulation signal *x*(*t*) and its components.

**Figure 10:**
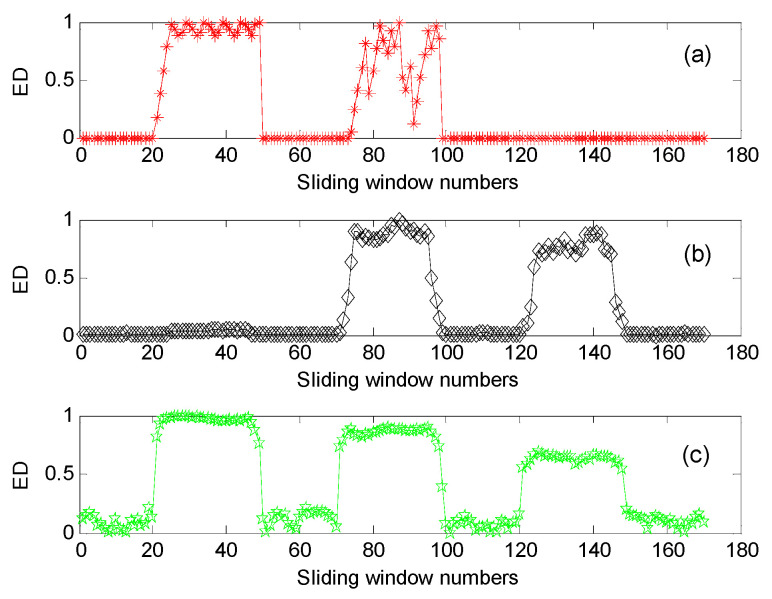
The calculation results of Euclidean distance obtained by different complexity methods for simulation signal *x*(*t*): (**a**) LZC, (**b**) MLZC and (**c**) GCMLZC.

For convenient comparison, we firstly used a sliding window with 80% overlap (i.e., the number of the overlapping data points is 800) to conduct the data interception along the simulation signal *x*(*t*), where the window width of the sliding window was 1000 data points, i.e., there were 171 sliding windows in total. Then, Euclidean distances (ED) of three complexity indexes (i.e., LZC, MLZC and GCMLZC) of the intercepted data were calculated to describe the complexity and uncertainty of the simulated signal *x*(*t*). The largest scale factors $\tau_{m}$ of the GCMLZC and MLZC were set as 20. Figure 10 shows the ED calculation results obtained by different complexity methods for the simulation signal *x*(*t*). It can clearly be seen from Figure 10 that LZC can only depict the changing state of the component *x*_1_(*t*) and *x*_2_(*t*) of the simulation signal *x*(*t*). In addition, the changing state of the component *x*_2_(*t*) and *x*_3_(*t*) of the simulation signal *x*(*t*) can be detected in MLZC, whereas the proposed GCMLZC can track the changing of all components of the simulation signal *x*(*t*). Furthermore, compared with LZC and MLZC, the Euclidian distance of the proposed GCMLZC has smaller fluctuation and higher accuracy at the component detection position of simulation signal *x*(*t*), which indicates that the GCMLZC method has better complexity assessment and fault feature extraction performance.

**Figure 11:**
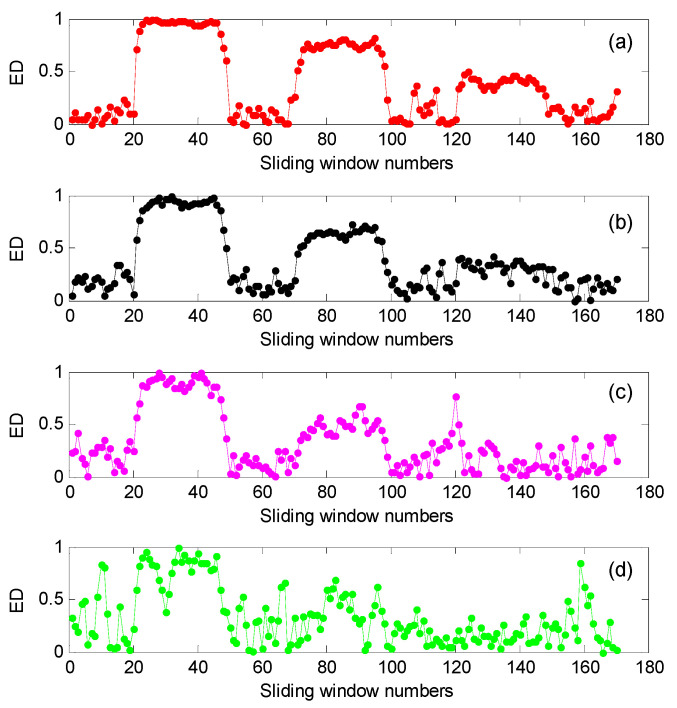
The calculation results of Euclidean distance obtained by the GCMLZC method for simulation signal *x*(*t*) under different noise levels: (**a**) SNR = 20 dB, (**b**) SNR = 15 dB, (**c**) SNR = 11 dB and (**d**) SNR = 10 dB.

To investigate the influence of the added signal noises on the performance of the GCMLZC method, we calculated the analysis results of the GCMLZC method under different noise levels (i.e., SNR = 20 dB, SNR = 15 dB, SNR = 11 dB and SNR = 10 dB), as shown in Figure 11. It can clearly be seen from Figure 11 that the complexity assessment ability of the GCMLZC method will be decreased with the decrease in SNR. When the SNR of the added signal noises is 10 dB, the GCMLZC method cannot accurately depict the complexity of simulation signal *x*(*t*). Therefore, the SNRs of the added signal noises are usually set to more than 10 dB, which is regarded as an empirical value.

## 4. The Proposed Fault Diagnosis Scheme

**Figure 12:**
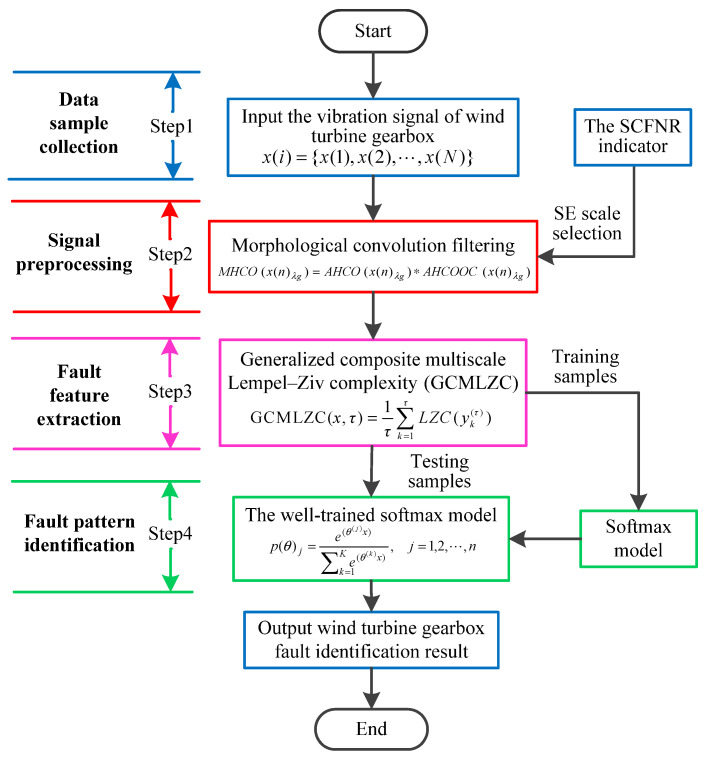
Flowchart of the proposed method for bearing fault identification.

To effectively identify wind turbine gearbox faults, this paper proposes a new intelligent fault diagnosis scheme based on MHCO and GCMLZC, which is mainly composed of four stages (i.e., data sample collection, signal preprocessing, fault feature extraction and fault pattern identification). Figure 12 shows the overall flowchart of the proposed fault diagnosis method, and its detailed procedure is expressed as follows:

Step 1: Data sample collection. Using the accelerometer to collect wind turbine gearbox vibration signals x(i)={x(1),x(2),⋯,x(N)}.

Step 2: Signal preprocessing. Morphological convolution filtering (i.e., MHCO) is adopted to preprocess the originally collected wind turbine gearbox vibration signal, which is aimed at weakening noise interference and highlighting fault features. Meanwhile, the SCFNR indicator is employed to select the optimal SE scale of MHCO.

Step 3: Fault feature extraction. According to the calculation process of GCMLZC, GCMLZC of the filtered signal is calculated to extract fault features of the wind turbine gearbox under different health conditions.

Step 4: Fault pattern identification. The extracted fault features are randomly divided into training samples and testing samples, where the training samples are used to train the softmax model and the testing samples are input into the well-trained softmax model to automatically identify wind turbine gearbox faults. In this step, the output of the softmax model is defined by

(26) $$p{\left(\theta \right)}_{j}=\frac{e^{\left(\theta^{\left(j\right)}x\right)}}{\sum_{k=1}^{K}e^{\left(\theta^{\left(k\right)}x\right)}}\begin{array}{cc} , & j=1,2,\cdots ,n \end{array}$$

where $p{\left(\theta \right)}_{j}$ represents the probability corresponding to the *j*th fault type, *K* is the number of fault types and θ denotes the parameters learned from the input samples.

## 5. Experimental and Engineering Application

### 5.1. Case 1: Experimental Gearbox Data Analysis

**Figure 13:**
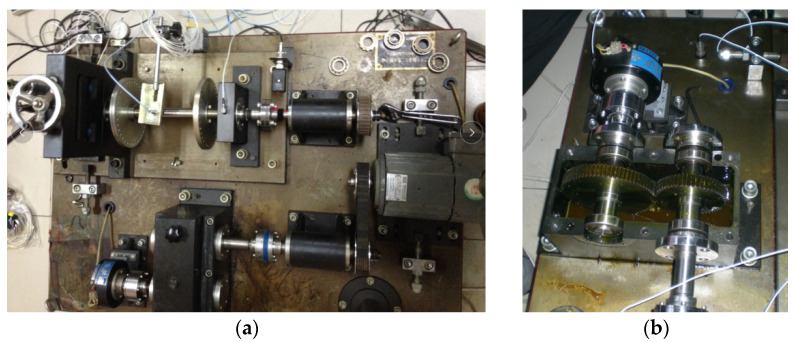
(**a**) Gear fault simulation experimental device and (**b**) gearbox structure drawing.

**Figure 14:**
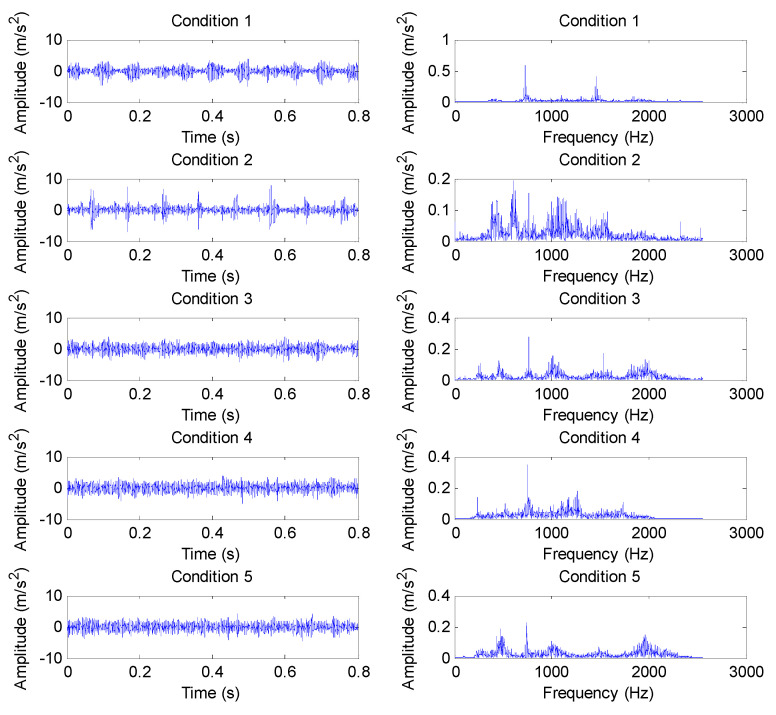
Time domain waveform and amplitude spectrum of gear vibration signal under different health conditions in case 1.

**Figure 15:**
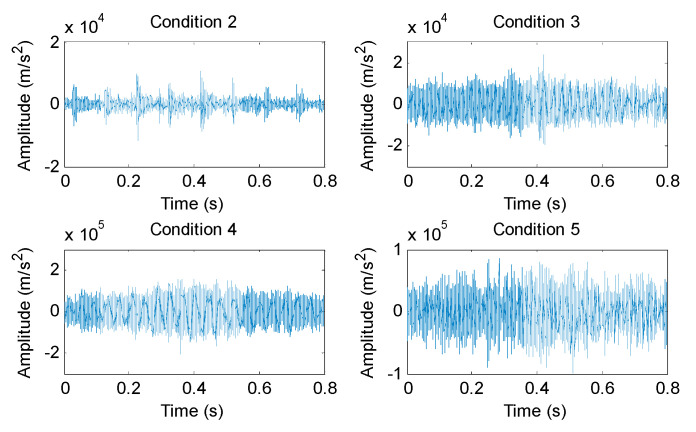
The filtered results obtained by MHCO for different experimental gear fault signals.

**Figure 16:**
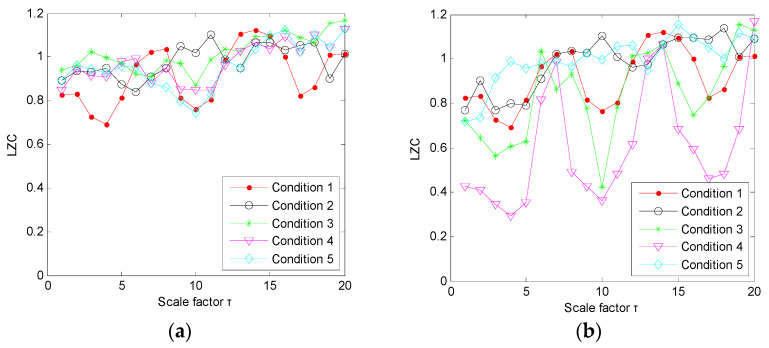
(**a**) GCMLZC of the original gear vibration signals and (**b**) GCMLZC of the filtered gear vibration signals in case 1.

**Figure 17:**
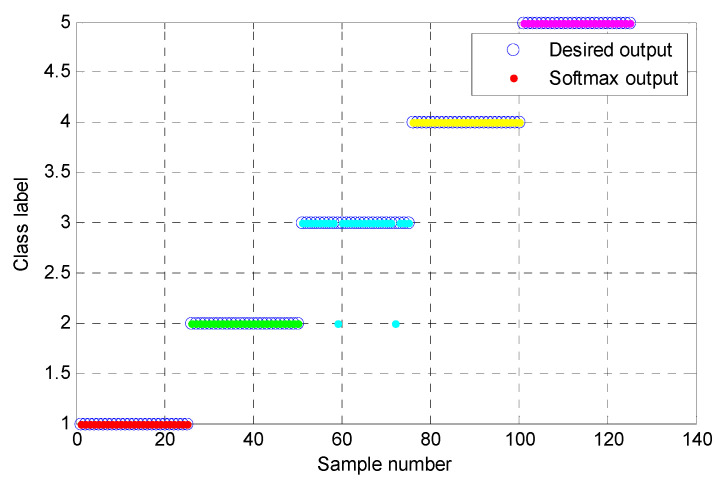
Identification results of the proposed method for gear datasets in the first trial.

**Figure 18:**
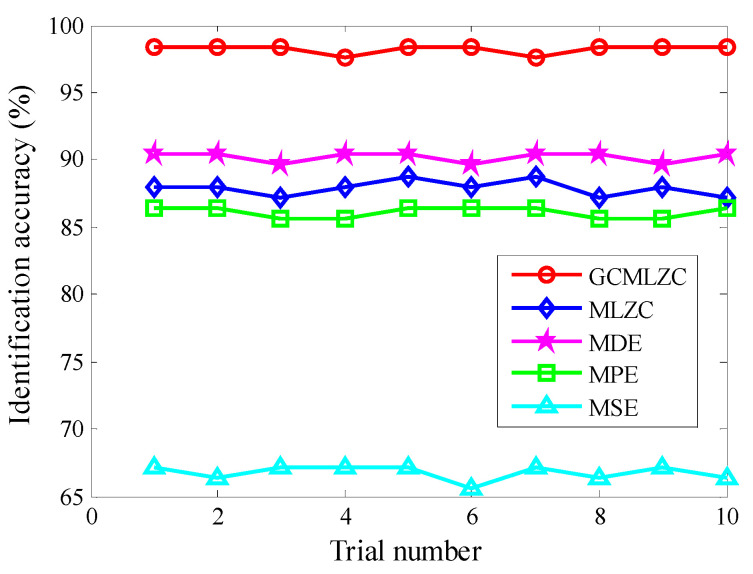
Identification accuracy obtained by different methods for 10 trials in case 1.

The proposed method was adopted to analyze gear vibration data collected from the laboratory of testing technology and fault diagnosis, North China Electric Power University (NCEPU). The experimental gear fault device was mainly composed of a driving motor, bearing, gearbox, shaft, turntable and governor. Figure 13a,b show photos of the experimental gear fault device and a gearbox structure drawing, respectively. In the experiment, gear vibration data were collected by using the accelerometer installed on the housing of the reduction gearbox with a sampling frequency of 5120 Hz. The experimental data collection system was mainly composed of an accelerometer, cable conductor, amphenol connector, signal conditioner, acquisition card and acquisition software, where the type of the data acquisition card was ADA16-8/2 (LPCI) with a single-terminal 8-channel input and 2-channel output. The motor speed could be adjusted by looking at the tachometer and turning the speed control knob. In addition, gear loading could be adjusted by switching on the brake and setting the level of braking torque. The specific steps can be found in the operating instructions of vibration analysis and the fault diagnosis test platform system for rotating machinery of QPZZ-II. The experimental gearbox was made up of two parts (i.e., the pinion and big gear). The pinion had 55 teeth, whereas the big gear had 75 teeth. In this experiment, the gearbox operated under five health conditions, including normal (condition 1), big gear pitting fault (condition 2), big gear fracture fault (condition 3), big gear pitting and pinion wear compound fault (condition 4), big gear fracture and pinion wear compound fault (condition 5). In addition, in this experiment, through speed adjustments, the motor operated at a rotating speed of about 800 rpm, but the actual speed and environmental interference under different health conditions differed somewhat, which indicates that the amplitude of the healthy condition may be greater than that of the unhealthy condition at a certain time point: the rotating frequencies of the small gear and big gear can be approximatively inferred as *f*_r1_ = 13.3 Hz and *f*_r2_ = 9.8 Hz, respectively. To verify the proposed method, 50 sets of gear vibration data under each health conditions were collected and each gear vibration signal consisted of 4096 data points. The training:testing data proportion was 1:1, i.e., the number of training samples and testing samples was the same, which was 125. Table 2 details the gear health conditions and sample selection. The time domain waveforms and amplitude spectra of gear vibration signals under different health conditions are shown in Figure 14. Notably, the plotted gear vibration signal belongs to the standardized results. The standardized formula is expressed as *x* = (*x* − mean(*x*))/std(*x*), where *x* is the collected original gear vibration signal, mean(*x*) is the mean value of *x* and std(*x*) is the standard deviation of *x*. As can be seen from Figure 14, the waveforms and spectra in different gear health conditions have certain similarities, especially for condition 3, condition 4 and condition 5, which implies that an effective method should be adopted to identify them. In order to facilitate the understanding, the identification performance of the proposed method was compared and analyzed from the following several aspects:

(1) The proposed method was utilized to analyze the collected gear vibration data. According to the flowchart of the proposed method, MHCO was first used to process different gear fault signals, where the optimal SE scale of MHCO was determined as 8 by using SCFNR. Notably, in this experimental data analysis, the search range of the flat SE scale λ was set as 1 to $\left\lfloor f_{s}/f_{r}\right\rfloor -2$, where *f*_s_ is the sampling frequency, *f_r_* is the rotating frequency of the input or output shaft and $\left\lfloor \cdot \right\rfloor$ denotes the down round operation. Due to the rotating frequency of the output shaft, *f*_r2_ = 9.8 Hz is smaller than that of the input shaft, i.e., when the maximum SE scale λ = $\left\lfloor f_{s}/f_{r2}\right\rfloor -2$, fault signatures of different gear health condition can all be covered. Hence, in experimental case 1, the flat SE scale λ=1,2,⋯,520. Figure 15 plots the filtered results obtained by MHCO for different gear fault signals. Subsequently, the GCMLZC of all data samples was calculated for fault feature extraction. For analysis, Figure 16a,b show the GCMLZC of one data sample for different gear health conditions before and after morphological convolution filtering, respectively. In the GCMLZC method, without a loss of generality, the largest scale factor $\tau_{m}$ is set as 20. As can be seen from Figure 16, GCMLZC with morphological convolution filtering has a better differentiation than GCMLZC without noise reduction. This proves the necessity of morphological convolution filtering in fault identification. Finally, the extracted GCMLZC was fed into the softmax classification model for automatically identifying different gear health conditions. Figure 17 shows the identification results of the proposed method for the first trial. Seen from Figure 17, the identification accuracy rate of the proposed method reached 98.4%, which indicates that only two data samples were misidentified; therefore, the proposed method is preliminarily proven to be effective in identifying gear fault types.

(2) To further verify the validity of the proposed method, comparisons among the proposed method and four representative complexity indexes (i.e., MLZC, multiscale dispersion entropy (MDE) [31], multiscale permutation entropy (MPE) [32] and multiscale sample entropy (MSE) [33]) were performed. To avoid randomness in the identification results of different methods and to ensure a fair comparison, all methods were preprocessed by the same morphological filtering (MHCO), the largest scale factor $\tau_{m}$ of all methods (i.e., GCMLZC, MLZC, MDE, MPE and MSE) were set as 20 and 10 trials were conducted. In addition, in the MDE method, the embedded dimension *m* = 3, time delay *d* = 1, the number of classes *c* = 5. In the MPE method, the embedded dimension *m* = 3 and time delay *d* = 1. In the MSE method, the embedded dimension *m* = 3 and the similarity tolerance r=0.15×SD, where *SD* is the standard deviation of the analyzed signal. Figure 18 shows the identification accuracies of different methods in 10 trials. In addition, Table 3 gives the detailed identification results of different methods, including the maximum, minimum and mean identification accuracy. Seen from Figure 18 and Table 3, the average identification accuracy (98.24%) of the proposed method was bigger than that of other methods (i.e., MLZC, MDE, MPE and MSE), whereas the standard deviation (0.3373) of the proposed method was lower than that of other methods, which means that the identification ability and stability of the proposed method are superior to other methods mentioned in this paper. Therefore, the effectiveness of the proposed method in gear fault identification is further validated by the above comparison.

(3) To consolidate the fault identification results, the fivefold cross-validation method was also applied to analyze the same gear vibration signal. Concretely, the data sample was first divided into five parts (each part had 50 samples), where four parts (i.e., 200 samples) were alternately regarded as the training samples and the remaining part (i.e., 50 samples) served as the testing sample. Next, five trials of different methods were performed, and the average identification accuracy values of five results were regarded as the ultimate identification accuracy of different methods. Table 4 gives the detailed diagnosis results obtained by different methods. As shown in Table 4, the proposed method achieved an average identification accuracy of 98.80%, whereas other complexity methods (i.e., MLZC, MDE, MPE and MSE) obtained 96.40%, 97.60%, 94.40% and 86.40% accuracy, respectively. The identification accuracy of the proposed method is clearly higher than that of other comparison methods. Consequently, the effectiveness and superiority of the proposed method is demonstrated once again.

### 5.2. Case 2: Engineering Data Analysis for Wind Turbine Gearbox

**Figure 19:**
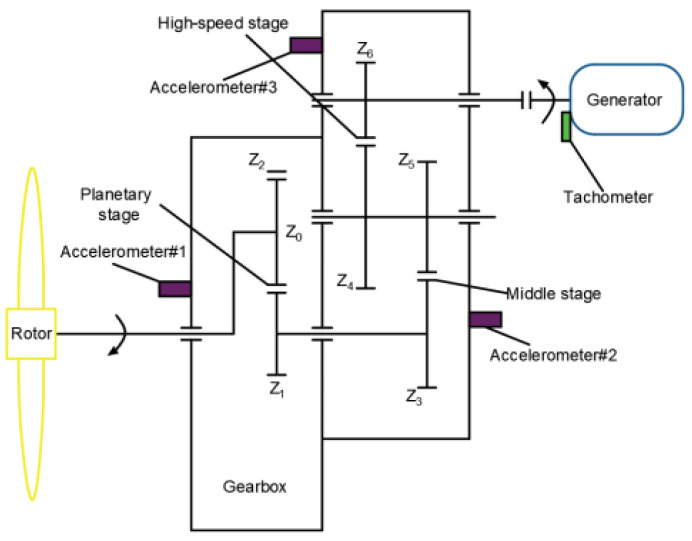
The structure diagram of wind turbine gearbox transmission system.

**Figure 20:**
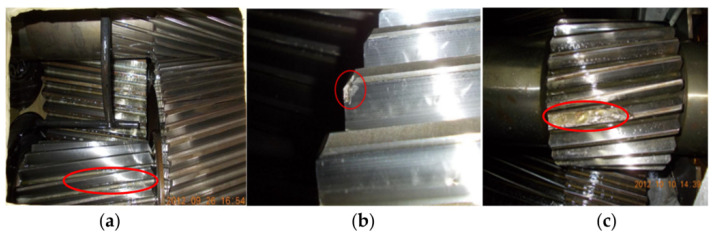
Three kinds of wind turbine gearbox faults: (**a**) pitting fault of small gear in middle stage, (**b**) spalling fault of big gear in high-speed stage and (**c**) fracture and wear compound fault of small gear in high-speed stage.

**Figure 21:**
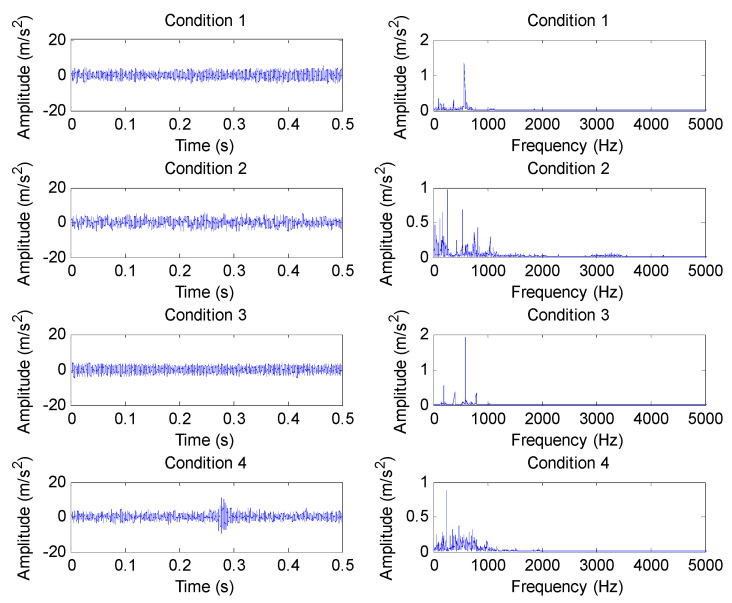
Time domain waveform and amplitude spectra of gear vibration data under different health conditions in case 2.

In this section, the proposed method was adopted to analyze the practical vibration data from a 1.5 MW wind turbine gearbox, which is located on a wind farm in northern China. Figure 19 shows a structural diagram of the wind turbine transmission system, which mainly consisted of a vane, spindle, rotor, gearbox and generator. The analyzed wind turbine gearbox adopted three-stage transmission (i.e., planetary stage, middle stage and high-speed stage), and was an FD1660 type. The rated power of the wind turbine gearbox was 1660 KW, and the weight of the gearbox was approximately 16,800 kg. In addition, the generator speed could be adjusted by using the electrical control system of the wind turbine. Table 5 lists the teeth numbers of each stage gear of the wind turbine gearbox, where Z_0_ denotes the teeth number of the planet gear, Z_1_ denotes the teeth number of the sun gear, Z_2_ represents the teeth number of the inner ring gear, Z_3_ and Z_5_ are the teeth numbers of the big gear and small gear in the middle stage, respectively, and Z_4_ and Z_6_ are the teeth numbers of the big gear and small gear in the high-speed stage, respectively. In engineering data analysis, gear vibration data were collected by an accelerometer (see Figure 19) glued onto the casing of the gearbox with a sampling frequency of 32,768 Hz. The wind turbine gearbox operated under four gear health conditions (i.e., normal, pitting fault of small gear in middle stage, spalling fault of big gear in high-speed stage, fracture and wear compound fault small gear in high-speed stage). When gear vibration data collection was conducted for each health condition, the wind speed was stable at about 12 m/s (corresponding to an input shaft speed of about 17 rpm and a power of about 1500 kW), and the speed of the high-speed shaft was stable at about 1400 rpm. Thus, the rotating frequencies of the high-speed shaft and middle shaft can be approximatively calculated as *f*_h_ = 23.33 Hz and *f*_m_ = 6.27 Hz, respectively. Figure 20 shows photographs of three gearbox faults. In the process of method validation, we obtained 100 data samples of each health condition. For each health condition, 50 samples were randomly selected as the training data, and the remainder was regarded as testing data. A total of 200 training and 200 testing samples were obtained, and each sample had 16,384 points. Apparently, it is a four-classification issue to be solved in essence. Table 6 presents detailed information of wind turbine gearbox data. Figure 21 shows the time domain waveform and amplitude spectrum of the gear vibration signal under different health conditions. Seen from Figure 21, gear fault conditions are difficult to be identified directly through observing the time domain waveform and amplitude spectrum, because different gear vibration data have certain self-similarity. Therefore, it is necessary to adopt an effective method to process the practical gearbox data.

**Figure 22:**
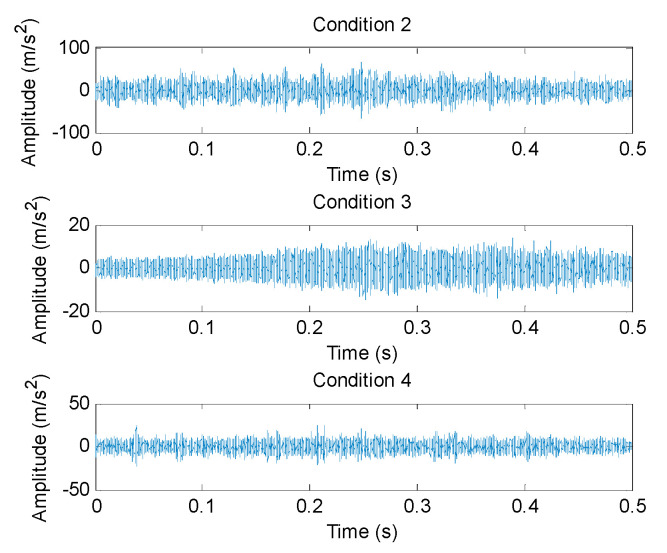
The filtered results obtained by MHCO for different practical gear fault signals.

**Figure 23:**
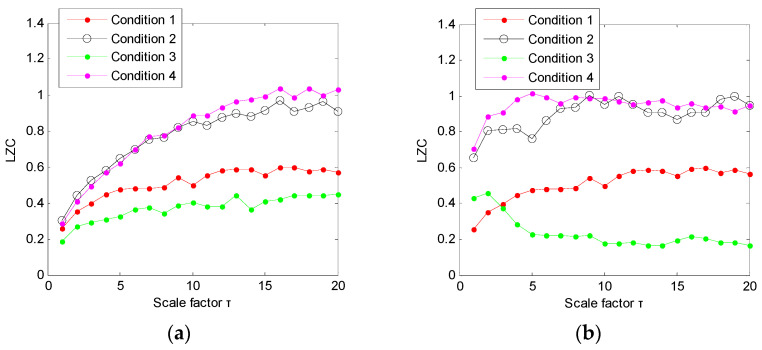
(**a**) GCMLZC of the original gear vibration signal and (**b**) GCMLZC of the filtered gear vibration signal in case 2.

**Figure 24:**
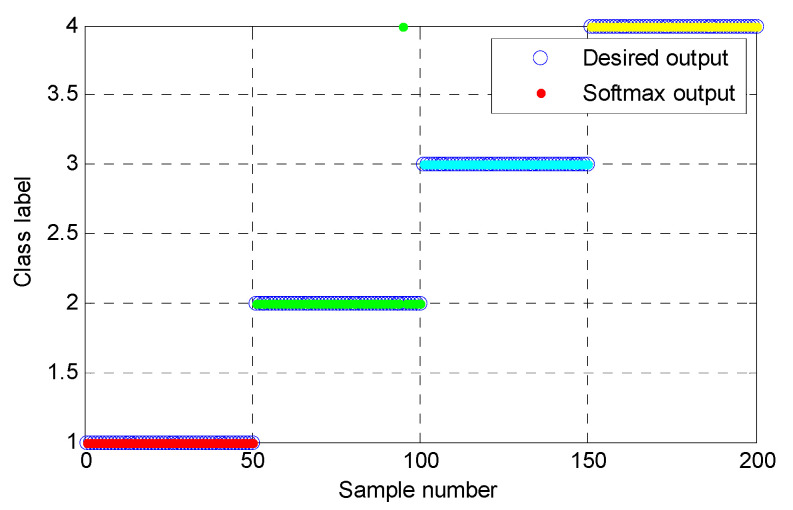
Identification results of the proposed method for the practical gearbox data in the first trial.

According to the flowchart in Figure 12, the proposed method was adopted to analyze the practical gearbox data. In the proposed method, based on the SCFNR indicator, the optimal SE scale of MHCO was selected as 10. Similar to case 1, the search range of the flat SE scale λ was set as 1 to $\left\lfloor f_{s}/f_{r}\right\rfloor -2$, where *f*_s_ is the sampling frequency, *f_r_* is the rotating frequency of the high-speed shaft or middle shaft and $\left\lfloor \cdot \right\rfloor$ denotes the down round operation. The rotating frequency of the middle shaft *f*_m_ = 6.27 Hz was smaller than that of the high-speed shaft, i.e., when the maximum SE scale λ = $\left\lfloor f_{s}/f_{m}\right\rfloor -2$, fault signatures of different gear health condition can all be covered. Hence, in experimental case 2, the flat SE scale λ=1,2,⋯,5224. Due to space limitations, the corresponding parameter optimization diagram is not included here. Figure 22 shows the filtered signals of three gear faults. For fault feature extraction, we calculated the GCMLZC of the filtered signal of all samples. Similar to case 1, in the GCMLZC method, the largest scale factor $\tau_{m}$ was selected as 20. Figure 23a,b show the GCMLZC of gear vibration signals before and after applying the MHCO method, respectively. As shown in Figure 23, after morphological convolution filtering, the degree of distinction of four gear health conditions is greater than that without filtering processing. This verifies the importance of morphological convolution filtering for signal preprocessing. Finally, the extracted GCMLZC was input into the softmax model for fault pattern identification. Figure 24 shows the identification results of the proposed method in the first trial. As shown in Figure 24, only one sample was misclassified, which indicates that the proposed method can obtain an identification accuracy of 99.5% (199/200). Thus, the proposed method exhibits good recognition performance for wind turbine gearbox faults.

**Figure 25:**
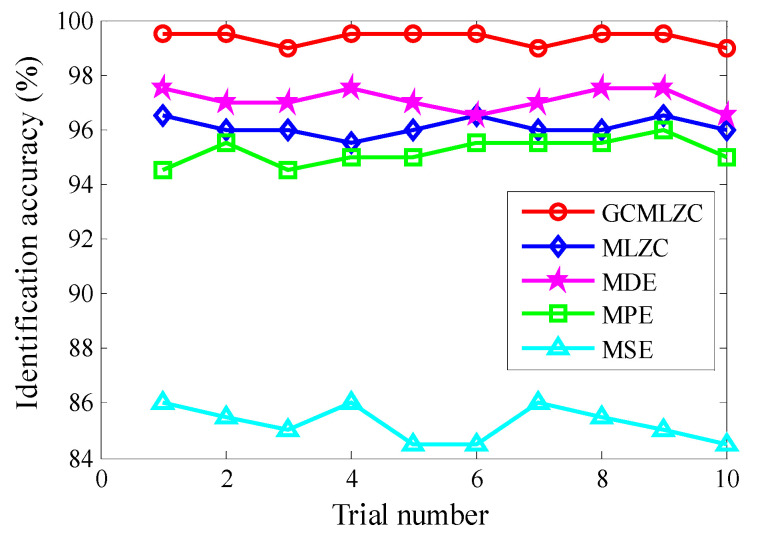
Identification accuracy obtained by different methods for 10 trials in case 2.

Similarly to case 1, to further prove the effectiveness of the proposed method, the identification abilities of five methods (i.e., GCMLZC, MLZC, MDE, MPE and MSE) were compared. Similarly, 10 trials of different methods were conducted to ensure the fairness of the comparison results. In addition, in all comparison methods (i.e., GCMLZC, MLZC, MDE, MPE and MSE), without a loss of generality, the largest scale factor $\tau_{m}$ was set as 20. In the MDE method, the embedded dimension *m* = 3, time delay *d* = 1 and the number of classes *c* = 5. In the MPE method, the embedded dimension *m* = 3 and time delay *d* = 1. In the MSE method, the embedded dimension *m* = 3 and the similarity tolerance r=0.15×SD, where *SD* is the standard deviation of the analyzed gear vibration signal. Figure 25 shows the fault identification accuracy of different methods in 10 trials, and Table 7 gives the detailed comparison results of different methods. Seen from Figure 25 and Table 7, the average identification accuracy (99.35%) of the proposed method was higher than that of other four methods (i.e., MLZC, MDE, MPE and MSE). In addition, standard deviation (0.2415) of the proposed method was less than that of other methods. This again indicates that the superiority of the proposed method in identifying wind turbine gearbox faults is verified.

To further consolidate the fault diagnosis results of wind turbine gearbox, we also used the fivefold cross-validation method to analyze the practical wind turbine gearbox data. Table 8 shows the detailed fault identification results obtained by different methods in the fivefold cross-validation. As can be seen from Table 8, the proposed method obtained an average identification accuracy of 99.50%, which is greater than that of the other comparison methods (i.e., MLZC, MDE, MPE and MSE). In other words, the proposed method has a stronger fault discriminant ability. This further proves that the proposed method is effective in extracting fault features from wind turbine gearboxes and identifying different gear fault categories.

### 5.3. Further Discussion

Although the proposed fault diagnosis scheme has been proven effective in identifying wind turbine gearbox faults, further research needs to be suggested. Firstly, in the signal preprocessing step of the proposed method, morphological convolution filtering can be replaced by other advanced techniques (e.g., local iterative filtering [34], total variation denoising [35] and sparse coding shrinkage), which is viewed as our future work. Secondly, in order to simultaneously obtain fault feature information of the vibration signal at different levels and scales, the idea of hierarchical decomposition can be integrated into the GCMLZC to further propose generalized hierarchical multiscale Lempel–Ziv complexity (GHMLZC), which is regarded as a future research direction. Thirdly, the softmax classification model was adopted in the fault pattern identification step of the proposed method; some other valuable classification models (e.g., weighted k-nearest neighbor, kernel extreme learning machine [36] and deep learning [37,38,39]) could also be adopted to replace the softmax model to automatically recognize wind turbine gearbox faults. Finally, the proposed method was only applied in gear fault diagnoses of wind turbines; therefore, our future work will focus on extending the proposed method to analyze other units (e.g., the bearing, rotor and blade) of mechanical systems. To avoid the dependence on knowing equipment information in advance, in future, we will integrate the proposed method into the recently popularized transfer learning model based on digital–analog drive to identify the unknown faults of different devices. In addition, it is worth mentioning that the proposed method was implemented on the MATLAB R2010a platform and operated on a computer with an Intel Core i7-9750H CPU @ 2.60 GHz/8.00 GB RAM processor. To implement and extend the proposed method repeatedly to other fields, the morphological filtering and Lempel–Ziv complexity software package will need to be downloaded, or the related code can be obtained directly from our research group.

## 6. Conclusions

In this paper, a new wind turbine gearbox fault identification method based on morphological convolution filtering and generalized composite multiscale Lempel–Ziv complexity has been presented. The main advantages of the proposed method are that fault feature extraction capability and identification accuracy can be improved by the combination of two methods (i.e., multiscale morphological-hat convolution operator and generalized composite multiscale Lempel–Ziv complexity). The effectiveness of the proposed method was also verified by experimental and engineering data analysis. Compared with traditional multiscale Lempel–Ziv complexity and several representative complexity indexes (i.e., multiscale dispersion complexity, multiscale permutation entropy and multiscale sample complexity), the proposed method could achieve a higher identification accuracy. Concretely, the contributions of this paper are summarized as follows:

(1) An effective noise reduction process, named a multiscale morphological-hat convolution operator, has been developed, which can solve the problem of the empirical selection of structuring elements with the aid of signal characteristic frequency-to-noise ratio;

(2) A complexity evaluation index, entitled generalized composite multiscale Lempel–Ziv complexity, has been proposed, which can avoid the problem of data length shortening appearing in multiscale Lempel–Ziv complexity;

(3) A new fault diagnosis scheme for wind turbine gearbox faults is proposed via the integration of a multiscale morphological-hat convolution operator and generalized composite multiscale Lempel–Ziv complexity;

(4) The experimental and engineering data analysis demonstrated the effectiveness of the proposed method in identifying wind turbine gearbox faults.

It should be pointed out that the influence of friction slip were not taken into account in the simulation model used in the paper. Hence, more accurate and comprehensive simulation signal analysis will be investigated in future research.

## Figures and Tables

**Table 1:** The largest SCFNR obtained by different methods and its corresponding optimal SE scale.

Different Methods	The Largest SCFNR Value	The Optimal SE Scale
GDE	0.5913	15
GCO	0.5661	18
GCOOC	0.5328	18
AHDE	0.3011	6
AHCO	0.2384	18
AHCOOC	0.2849	40
MGCO	2.5142	29
MHCO	57.8993	8

**Table 2:** The detailed description of gear datasets in case 1.

Condition Label	Gear State	Number of Training Samples	Number of Testing Samples	Class Labels
Condition 1	Normal	25	25	1
Condition 2	Gear pitting	25	25	2
Condition 3	Gear fracture	25	25	3
Condition 4	Gear pitting and wear	25	25	4
Condition 5	Gear fracture and wear	25	25	5

**Table 3:** Specific fault identification results of different methods in case 1.

Different Methods	Identification Accuracy Obtained Using Different Methods (%)			
	Maximum	Minimum	Mean	Standard Deviation
GCMLZC	98.40	97.60	98.24	0.3373
MLZC	88.80	87.20	87.92	0.5903
MDE	90.40	89.60	90.16	0.3864
MPE	86.40	85.60	86.08	0.4131
MSE	67.20	65.60	66.80	0.5657

**Table 4:** Fault identification results of different methods with fivefold cross validation in case 1.

Different Methods	Diagnosis Accuracy Obtained by Different Methods for Five Trials (%)					Average Accuracy (%)
	1	2	3	4	5	
GCMLZC	100	100	98.00	98.00	98.00	98.80
MLZC	96.00	98.00	96.00	96.00	96.00	96.40
MDE	98.00	96.00	98.00	98.00	98.00	97.60
MPE	94.00	94.00	96.00	94.00	94.00	94.40
MSE	86.00	88.00	84.00	86.00	88.00	86.40

**Table 5:** The teeth number of each stage gear of wind turbine gearbox.

Name	Planetary Stage			Middle Stage		High-Speed Stage	
	Z_0_	Z_1_	Z_2_	Z_3_	Z_5_	Z_4_	Z_6_
Teeth number	40	21	102	100	23	93	25

**Table 6:** Detailed description of wind turbine gearbox datasets in case 2.

Condition Label	Gearbox State	Number of Training Samples	Number of Testing Samples	Class Labels
Condition 1	Normal	50	50	1
Condition 2	Gear pitting fault	50	50	2
Condition 3	Gear spalling fault	50	50	3
Condition 4	Gear fracture and wear fault	50	50	4

**Table 7:** Specific fault identification results of different methods in case 2.

Different Methods	Diagnostic Accuracy Obtained Using Different Methods (%)			
	Maximum	Minimum	Mean	Standard Deviation
GCMLZC	99.50	99.00	99.35	0.2415
MLZC	96.50	95.50	96.10	0.3162
MDE	97.50	96.50	97.10	0.3944
MPE	96.00	94.50	95.20	0.4830
MSE	86.00	84.50	85.25	0.6346

**Table 8:** Fault identification results of different methods with fivefold cross validation in case 2.

Different Methods	Diagnosis Accuracy Obtained by Different Methods for Five Trials (%)					Average Accuracy (%)
	1	2	3	4	5	
GCMLZC	98.75	100	100	98.75	100	99.50
MLZC	95.00	96.25	95.00	93.75	95.00	95.00
MDE	97.50	98.75	97.50	97.50	98.75	98.00
MPE	96.25	96.25	98.75	96.25	96.25	96.75
MSE	87.50	86.25	86.25	87.50	85.00	86.50

## Data Availability

The data used in this study are all owned by the research group and will not be communicated.
